# Sex differences in judging self-orientation: the morphological horizon and body pitch

**DOI:** 10.1186/1471-2202-8-6

**Published:** 2007-01-07

**Authors:** Luc Tremblay, Digby Elliott

**Affiliations:** 1 University of Toronto, 55 Harbord Street, Toronto, Ontario, M5S 2W6, Canada; 2 McMaster University, 1280 Main Street West, Hamilton, Ontario, L8S 4K1, Canada

## Abstract

**Background:**

Sex differences exist for many spatial tasks. This is true for circular vection, field dependence, and perception of veridical vertical with body tilt. However, explanations for these sex differences is lacking in the literature. In this study, we investigated the nature of individual differences in the perception of self-orientation in humans. Male and female participants were asked to identify their Morphological Horizon (i.e., line perpendicular to saggital plane at eye-level) in different body orientations relative to gravity (i.e., 45 deg and 135 deg body pitch) with and without prior whole body rotation.

**Results:**

Sex explained the observed differences in the perception of self-orientation only when blood distribution was least altered (i.e., 45 deg body pitch) and without prior whole body rotation. Specifically, females presented a more footward bias than males in these conditions.

**Conclusion:**

These results add to the literature on sex differences for spatial orientation tasks. As the differences were only observed with static conditions and when blood distribution was least affected, we concluded that sex differences in the perception of self-orientation are associated with gravireceptors (e.g., otoliths).

## Background

The perception of spatial orientation is influenced by visual information and the inertial forces that affect our body. For example, the perception of true vertical is biased by a tilted visual environment [1,2] and by variations in the direction and/or magnitude of inertial forces acting on the body [3-7]. However, such perceptual biases present with altered sensory stimulation are not consistent across sexes.

Witkin and colleagues reported sex differences in the perception of verticality using the Rod-and-Frame Test (RFT). The RFT requires the participant to orient a rod to the perceived vertical with and without a tilted surrounding frame. Asch and Witkin [1] observed that "the perceived upright was always much closer to the visual than the postural vertical" (p.335). The perceptual bias was explained by a multisensory affordance of body tilt when the visual environment is tilted and was termed field dependence. Interestingly, females are more field-dependent than males [8]. This influence of a visuo-spatial reference also affects females to a greater extent than males when assessed with direct vestibular stimulation using body tilt [9]. Further, males perform differently than females for many other spatial tasks (see [10] for a meta-analysis; see also [11]). In sum, males and females integrate afferent information related to spatial orientation differently [12]. However, these empirical observations benefit from very few explanations [13].

Reinking *et al*. [14] demonstrated that instructing females to pay attention to internal sensory cues reduced their frame dependency in the RFT. Thus, cognitive processes significantly affect the perception of spatial orientation (PSO). More recently, we tested this cognitive explanation by asking males and females to identify their Morphological Horizon (MH; i.e., place a single point of light in an otherwise dark environment at the perceived straight-ahead at eye level) in different body pitch orientations while either receiving no attentional instructions, instructions to pay attention to external sensory cues, or instructions to pay attention to internal sensory cues [15]. Although females were affected by the instructions (i.e., least biased when paying attention to internal cues), sex differences were still significant in all conditions. These results provided some indirect support for an anatomical explanation (see also [16]).

In males, the utricle, saccule and superior semi-circular canals are significantly larger than in females [17]. As such, it is possible that anatomical differences in the vestibular apparatus between males and females are associated with sex differences in the perception of spatial orientation. If this is the case, then whole body rotation and/or altered body orientation should affect the PSO differently in females and males.

The present study was designed to determine what type of body orientation manipulations mediate sex difference in the PSO with the goal of identifying the type of receptor involved. Perceived morphological horizon (PMH) was measured immediately after a whole body rotation (i.e., superior semi-circular canal) or while remaining stable in the same orientation (i.e., otoliths), and in different body pitch orientations (i.e., 45° and 135° supine) (i.e., baroreceptors). In accordance to previous literature, we anticipated that sex differences in PMH would be observed only when the body orientation is altered, stable, and with the least amount of blood redistribution (i.e., 45° supine).

## Results

### 45° orientation

**Figure 1:**
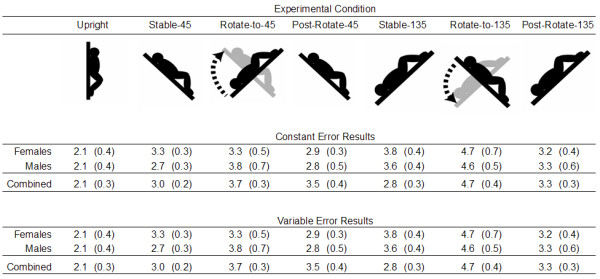
Constant and Variable PMH error data (deg) with standard error for all Conditions.

**Figure 2:**
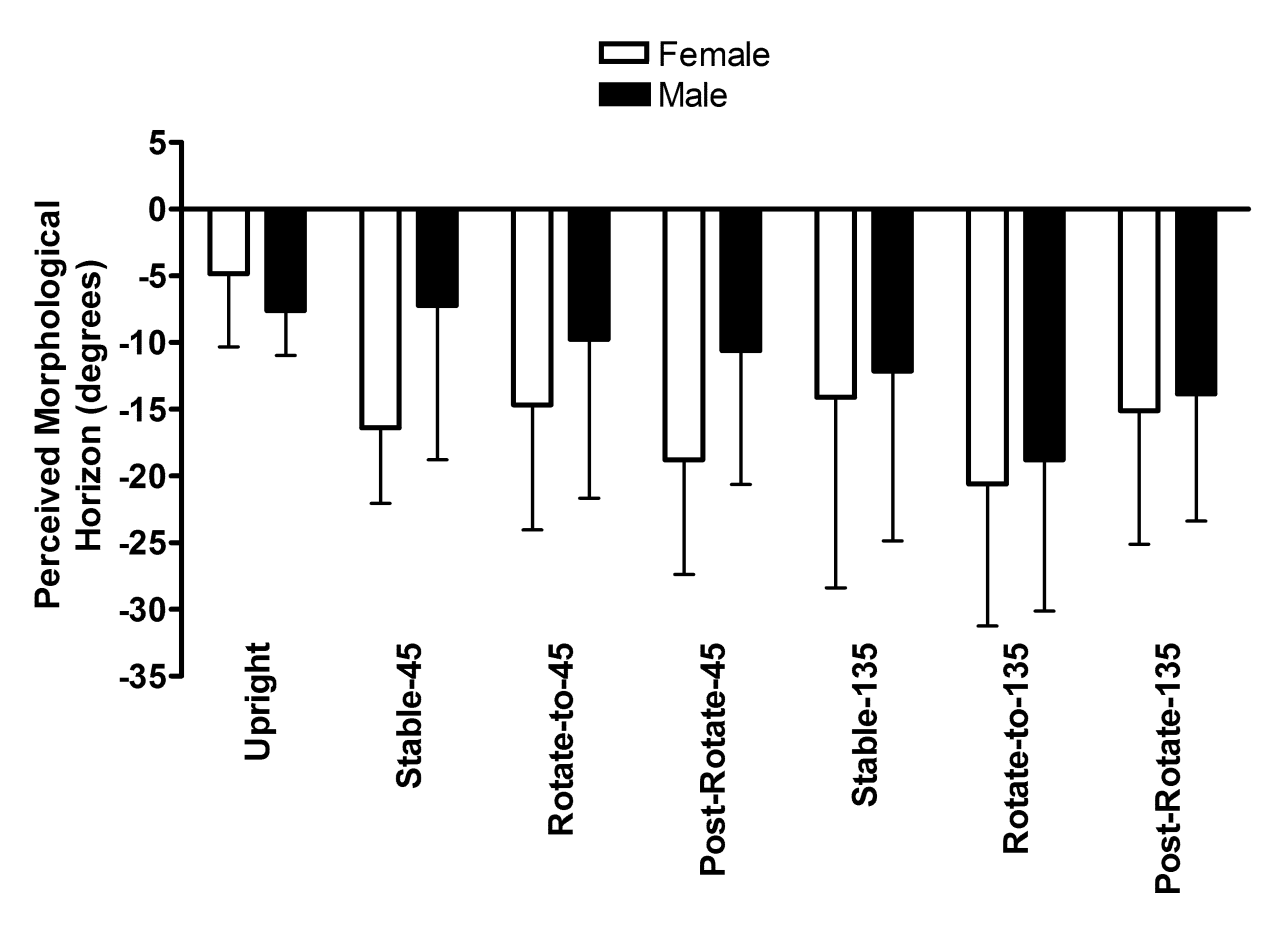
Constant PMH error (deg) with standard error as a function of Sex and Condition.

The constant error in PMH analyses for the 45° body pitch conditions yielded a main effect for Condition, *F *(3, 51) = 6.63, *p *< .001 and an interaction between Sex and Condition, *F *(3, 51) = 3.82, *p *< .05 (see Figure 1). Overall, participants presented a more footward bias in the Stable-45, the Rotate-to-45, and the Post-Rotate-45 conditions than in the Upright condition. However, the breakdown of the Sex by Condition interaction revealed that this effect was only true for the females participants (see Figure 2).

For the variable error analyses in PMH for the 45° orientation conditions, there was an effect of Condition, *F *(3, 51) = 5.39, *p *< .01, which revealed that individuals were more variable immediately after a whole body rotation condition (i.e., Rotate-to-45) than in the Upright condition (see Figure 1).

The trial by trial analysis was also significant, *F *(9, 153) = 6.91, *p *< .001. Specifically, participants showed less footward bias in the first trial than in trials five to ten. Likewise, there was a smaller footward bias in the second and the third trial than in trials eight to ten (see Table 1).

### 135° orientation

The constant error analysis for the 135° body pitch conditions yielded a main effect for Condition, *F *(3, 51) = 14.13, *p *< .001 but no effect or interaction involved Sex. As depicted in Figure 2, the PMH was less biased in the Upright condition than in all other conditions. As well, PMH was more biased in the Rotate-to-135 condition than in the Stable-135 and the Post-Rotate-135 conditions.

The variable error analysis for the 135° conditions revealed a main effect for Condition as in the 45° orientation conditions, *F *(3, 51) = 12.28, *p *< .001. Post hoc analysis of this effect revealed that participants more variable immediately after a whole body rotation condition (i.e., Rotate-to-135) than in both the Post-Rotate-135 and Upright conditions. As well, participants were less variable in the Upright condition than in all 135° orientation conditions.

**Figure 3:**
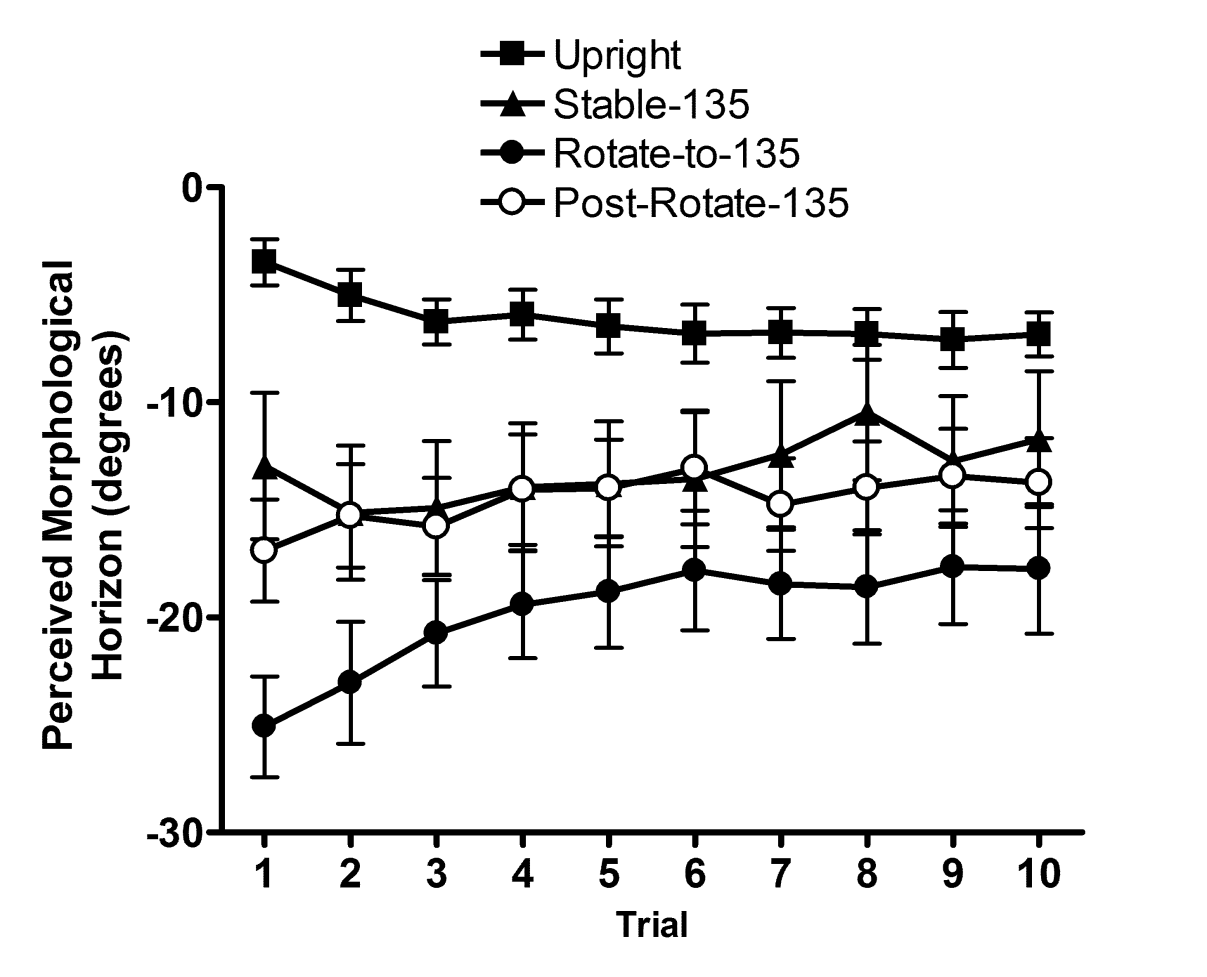
Constant PMH error (deg) with standard error as a function of Trial for 135° conditions.

Concerning the trial by trial analysis for the 135° conditions, a main effect for Trial, *F *(9, 153) = 3.54, *p *< .001, as well as a Condition by Trial interaction, *F *(27, 459) = 4.21, *p *< .001, were observed. Specifically, there were greater footward biases in trials one and two than in trials eight and ten (see Table 1). As well, the post hoc analysis of the Condition by Trial interaction revealed a greater footward bias in the second and third trial than in the eighth trial of the Stable-135 condition (see Figure 3). Finally, for the Rotate-to-135 condition, we observed a greater footward bias in the first than in the fourth to the last trial, and also a greater footward bias in the second than in the sixth to the last trial (see Table 1 and Figure 3).

### Correlational analyses

Correlation analyses were conducted to determine the relative contribution of sex and three indices of blood volume on PMH. These indices were weight, height, and body mass index. Body mass index is calculated by dividing the mass by the squared height of the participant. On average, females were 1.7 m tall, weighed 61 kg, which yielded an average body mass index of 21.3 kg/m^2 ^and males were 1.8 m tall, weighed 73 kg for an average body mass index of 22.3 kg/m^2^. In these analyses, we used weight, height, and body mass index to predict PMH bias from upright for two blocks of five (5) trials in each condition (i.e., difference between average error in the experimental condition and the upright condition).

As would be expected from the inferential statistics, sex accounted for a significant amount of variance (*p *< .05) in PMH in both blocks of trials of the Stable-45 and the Post-Rotate-45 conditions (Stable-45: Block 1, *r*_*b *_= .57, Block 2, *r*_*b *_= .63; Post-Rotate-45: Block 1, *r*_*b *_= .54, Block 2, *r*_*b *_= .57). More specifically, females presented a greater footward bias than males. Sex did not have a significant impact on perceptual judgements in any of the other conditions. Moreover, sex differences at Stable-45 and Post-Rotate-45 were independent of all blood volume indices.

Interestingly, while our indices of blood volume failed to explain the obtained sex differences in the 45° conditions, body mass index and perceptual bias were significantly correlated in the second block of the Rotate-to-135 (i.e., 135° orientation) (*r *= .48). Note that it is in the Rotate-to-135 condition that both males and females exhibited the largest footward bias. The relationship between body mass index and PMH indicates that people with lower body mass index present larger footward bias than people with higher body mass index. Thus, while the shifts in blood volume were sufficient to create an important perceptual bias in the 135° conditions, they did not contribute significantly to the observed sex effects found in the 45° conditions. Indeed, when body mass index, weight and height were included as covariates in the 45° conditions analyses of variance, they accounted for 6.2%, 0.71%, and 4.15% of the between-participant variability, respectively, and they accounted for 13.4%, 0.27%, and 5.9% of the between-participant variability, respectively, in the ANOVA for the 135° conditions.

## Discussion

Firstly, all experimental conditions in the 45° and 135° orientations significantly affected PMH as compared to the upright condition. The typical perceptual bias is footward and is analogous to the elevator illusion, which is explained by the variation of inertial forces acting on the otoliths [7,18] although not fully explained by variation in eye position [5].

As hypothesized, some effects were not different between males and females. For instance, the effect of Rotate-to-135 changed as a function of trial, but that was similar for both sexes. This significant effect of body rotation on PMH for both males and females may have arisen from superior semi-circular canal stimulation (or other sources of information stimulated during whole body rotation; see [19]). Our results also support the idea that blood distribution in the body influences the perception of self-orientation [20,21]. Indeed, the 135° conditions (i.e., Stable-135, Rotate-to-135, and Post-Rotate-135) induced more blood redistribution (i.e., more baroreceptors stimulation) than the 45° conditions and led to significant footward biases both for females and males. Further, this blood redistribution also affected PMH variability as participants exhibited a smaller variable error in the Upright condition than in both conditions immediately following a whole body rotation (i.e., Rotate-to-45 and Rotate-to-135). On the other hand, it was particularly when participants were in the stable conditions of the 45° pitch position that sex differences were observed.

Females demonstrated a footward bias in PMH when submitted to the 45° conditions (i.e., Pitch 45, Rotate-to-45, and Post-Rotate-45) as compared to the Upright condition. Conversely, males were not affected by the sole modification of the direction of inertial forces. Although we did not expect sex differences in PMH in the Rotate-to-45 condition, the correlational analyses for the 45° conditions fully supported our predictions. Indeed, sex explained a significant amount of variance of the PMH biases in the stable 45° body pitch conditions only (i.e., Stable-45 and Post-Rotate-45). These findings indicate that variations in the direction of inertial forces are associated with sex differences in the PMH. Certainly, further investigations in the perception of self-orientation are required to better explain the observed individual differences and further test our anatomical difference explanation [15]. At the very least, sex differences appear to be associated with the integration of information from gravireceptors or statoliths. Further, it is important to note that our explanation assumes that the observed results arise from visual-vestibular interactions. Indeed, it is important to note that identifying the perceived upright and horizon may not involve the vestibular system at all. Although it is difficult to explain why only stable altered body orientations yielded significant differences between females and males without consideration for the vestibular input, this perspective must be mentioned. Nevertheless, as compared to this purely visual explanation, our empirical observations yielded more support to our hypothesis considering visual-vestibular interactions.

In this study, we found significant sex differences in PMH. However, this does not mean that all males are immune to the perceptual effects induced by variation in the direction of inertial forces. Indeed, collapsing across trials in the Stable-45 condition revealed greater between-subject variability for males than for females (*Fmax *(8, 9) > 6, *p *< .05). Thus, stating that the perception of self-orientation – under visually deprived environment and altered vestibular stimulation (i.e., true or afforded by the visual stimuli) – is less biased for males than for females is misleading. Indeed, if the observed sex differences in this type of work stem from of anatomical differences (e.g., size of the otoliths; [17]), then these anatomical differences should more accurately predict the perception of self-orientation than sex. From this perspective, it may be appropriate to consider specific individual differences other than sex per se in future research on the perception of spatial orientation.

## Conclusion

Sex differences have been found for many spatial tasks [10,22]. Our findings are consistent with studies on circular vection [23-25], field dependence [8], and perception of verticality with body tilt [9]. Indeed, without significant blood redistribution and when whole body rotation did not immediately precede a perceptual judgment, sex differences in the perception of spatial orientation were observed. As such, sex differences in the perception of self-orientation appear to be associated with sources of gravireception. It has been suggested that anatomical differences explain the perceptual differences [15,16] and there are sex differences in the size of the otoliths [17]. However, many other sources of gravireception must be assessed (e.g., neck proprioception; see [26]) before fully understanding the sensory contributions to sex differences in the perception of spatial orientation.

## Methods

### Participants

In this study, 20 members of the McMaster community (10 females, 10 males) participated in exchange for a financial compensation ($5). All participants had normal or corrected-to-normal vision and were unaware of the goal of the experiment. The mean age of the group was 21.6 years old (SD = 2.3 years) and all participants but three males and one female participant were right-handed.

### Apparatus

**Figure 4:**
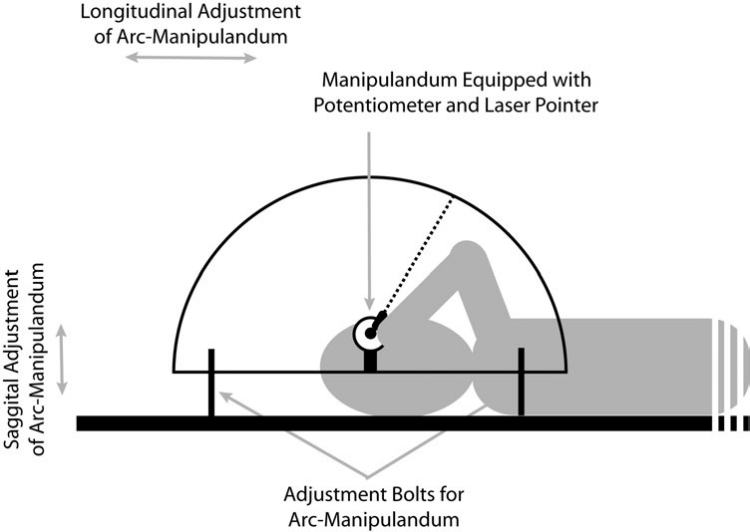
Side view illustration of the arc-manipulandum and participant on the body pitch device.

The equipment consisted of a MH perception device and a body pitch device located in a dark room. The MH perception device first consisted of an arc with a radius of 53.5 cm. In the center, we placed a round smooth manipulandum equipped with a potentiometer and a laser pointer (see Figure 4). The arc was made of a 4.5 cm wide piece of polymer painted in black. The potentiometer was linked through an Analog-to-Digital converter (Dataq DI-220, Dataq Instruments Inc., Akron, OH, USA) to a PC, which acquired data with the Windaq program (Dataq Instruments Inc.). The potentiometer input was sampled at 100 Hz and provided a spatial resolution of 0.08 degrees. The manipulandum allowed the participants to adjust the position of the laser beam at PMH on the arc in a self-paced manner. This arc-manipulandum was installed on a 7 cm by 107 cm piece of plywood that could be moved along the saggital and longitudinal axis of the participant in such a way that the shaft of the potentiometer could be centered with the participant's eyes. This MH perception device was attached to the body pitch device on the right-hand side of the participant (see Figure 4). The dark room allowed us to limit visual information to the single laser dot on the arc of the MH perception device.

The body pitch device consisted of a bed rotating around its transverse axis, which was installed on a supporting frame secured to the wall. The participant was secured on the bed by the means of a waist harness (KBoum: Gymnova, Laval, QC, Canada) and a set of carabiners. Spine board straps (Ferno Canada, Mississauga, ON, Canada) were used to stabilize the shoulders and a pair of Velcro straps was used for the feet. An adjustable cervical immobilization collar (Wizloc: Ferno Canada) restricted head movements. The bed was equipment with an 8 cm thick foam mattress for comfort. Two pairs of straps were used to maintain the bed in the appropriate positions.

### Task & procedures

This research has been performed in accordance to the ethical standars set in the 1964 Declaration of Helsinki. Before the experimental session, the participant provided informed consent according to the rules and guidelines of the ethics board of McMaster University. Afterward, the participant was secured to the bed with the above-mentioned pieces of equipment. Two experimenters were required for the experimental sessions. One experimenter manipulated the participant's body orientation by adjusting the bed while the other experimenter collected perceived morphological horizon data.

A typical trial was as follows. One experimenter oriented the laser of the manipulandum in a random position. From trial to trial, this position was alternated between upward and downward from objective morphological horizon. Then, the same experimenter gave a "GO" signal to the participant. At that point, the participant was allowed to open her/his eyes and place the laser beam at the PMH. Participants were also asked to keep their eyes still during each trial. The laser beam on the arc was the only source of visual information in an otherwise dark environment. Indeed, we insured that participants could only perceive a single point of light in space during the trials, without any other visual reference available to them during the experiment [27]. The participants were asked to complete their response within 5 s. When the response was completed, the participant gave an "OK" signal. On that signal, the second experimenter triggered a marker on the data collection program. At the same time, participants closed their eyes in preparation for the next trial.

**Figure 5:**
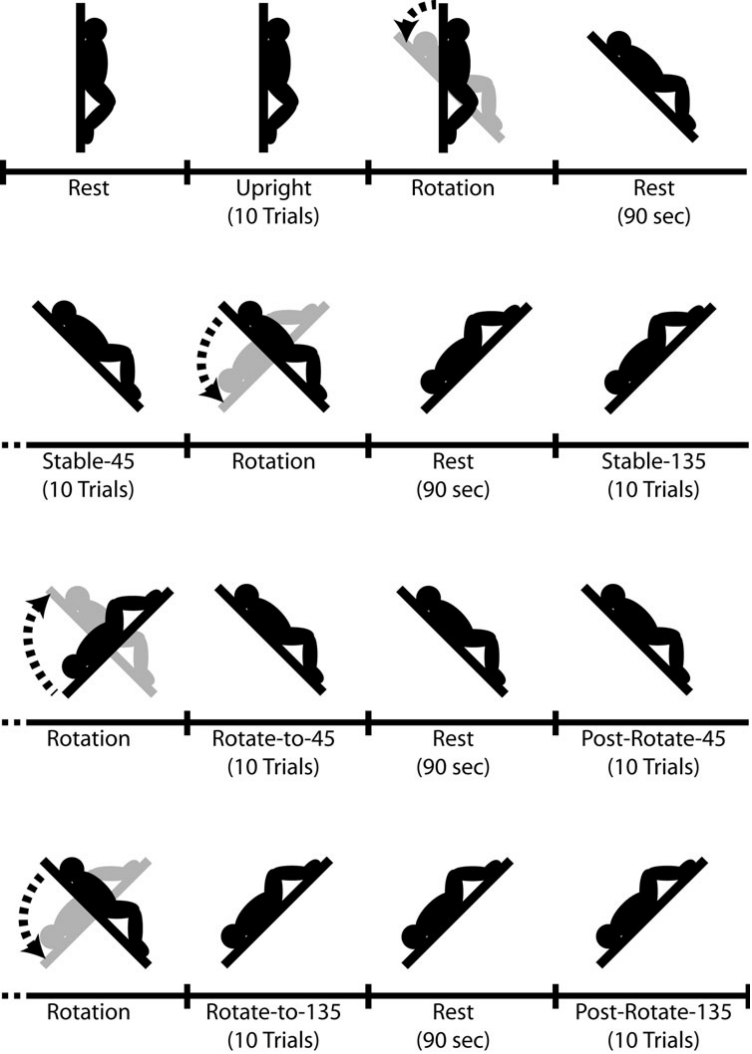
Illustration of the experimental conditions.

All participants were first placed in vertical upright orientation for 10 trials. Then, participants were asked to perform 10 trials at the 45° and 135° orientations preceded by a 90 s period (i.e., Stable-45 and Stable-135). It is important to note that only supine (i.e., backward) pitch orientations were used in this experiment. The starting position was counterbalanced across participants but only the start with Stable-45 condition is described here (see Figure 5).

After the Stable-45 and Stable-135 conditions, participants were rotated to the 45° orientation and asked to immediately perform 10 trials (Rotate-to-45). This condition was followed by a 90 s waiting period followed by another 10 trials in the same orientation (Post-Rotate-45). Then, the same sequence was performed going to the 135° position (Rotate-to-135 and Post-Rotate-135).

Altogether, the set of conditions combined PMH measurements with and without whole body rotation prior to the trials. This occurred both without (45° conditions) and with (135° conditions) unusual blood distribution [28]. As well, these conditions controlled for any potential after-effect of whole body rotation as PMH performance was measured following a 90 s delay, both before and after a whole-body rotation (i.e., Stable-45 and Post-Rotate-45, as well as Stable-135 and Post-Rotate-135).

As the literature on sex differences in spatial orientation tasks does not typically involve whole body rotations and unusual blood distribution, we hypothesized that sex differences in PMH would be observed only in the Stable-45 and Post-Rotate-45 conditions. Such a result would provide some support for our "hardware" hypothesis, which stipulates that sex differences in the perception of spatial orientation stem from differences in utilizing afferent information from the otoliths [15].

### Analyses

We calculated the constant error (CE) and variable error (VE) in degrees from the spatial error measures and performed separate analyses of variance for each experimental orientation (i.e., 45° and 135°). Thus, separate 2 Sex (Male, Female) by 4 Condition (Upright, Pitch, Rotation, and Post-Rotation) mixed analyses of variance were conducted. As well, in order to test for trial to trial adaptation, we also performed two separate 2 Sex by 4 Condition by 10 Trials (1–10) mixed analyses of variance on the error observed on each trial. For an unknown reason, the cervical collar was not properly fitted to one of the males and moved during testing. Not surprisingly, the data of that participant presented variability greater than two standard deviations from the average variability of the group and were removed from the analyses.

## Authors' contributions

LT and DE participated in the design of the study, performed statistical analyses, and worked on the drafting of the manuscript. Only LT performed data collection and reduction. All authors read and approved the final manuscript.

## Figures and Tables

**Table 1:** Perceived Morphological Horizon in Degrees (and Standard Error) as a Function of Trial and Condition

	Trial									
	
Condition	1	2	3	4	5	6	7	8	9	10
Upright	-3.5 (1.1)	-5.0(1.2)	-6.3 (1.0)	-5.9 (1.2)	-6.5(1.3)	-6.8 (1.4)	-6.8 (1.2)	-6.8 (1.2)	-7.1 (1.3)	-6.8 (1.0)
Stable-45	-11.6 (2.0)	-10.9 (2.2)	-11.2 (2.4)	-11.9 (2.4)	-11.5(2.5)	-11.5 (2.3)	-12.2 (2.4)	-13.1 (2.3)	-13.1 (2.5)	-13.4 (2.6)
Rotate-to-45	-10.1 (1.8)	-11.2 (2.3)	-11.4 (2.2)	-11.2 (2.7)	-12.7 (2.9)	-11.8 (3.0)	-13.2 (2.6)	-13.5 (2.6)	-14.3 (2.6)	-14.1 (2.8)
Post -Rotate-45	-12.5 (2.2)	-14.2 (2.4)	-13.0 (2.6)	-15.5 (2.5)	-15.1 (2.3)	-16.8 (2.2)	-15.1 (2.5)	-15.3 (2.4)	-15.4 (2.3)	-16.1 (2.2)
Stable-135	-13.0(3.4)	-15.1 (3.1)	-14.9 (3.1)	-13.9 (3.0)	-13.8 (2.9)	-13.6 (3.2)	-12.4 (3.4)	-10.5 (3.2)	-12.7 (3.0)	-11.7 (3.2)
Rotate-to-135	-25.1 (2.3)	-23.0 (2.8)	-20.7 (2.5)	-19.4 (2.5)	-18.8 (2.6)	-17.8 (2.8)	-18.5 (2.5)	-18.6 (2.6)	-17.7 (2.6)	-17.7 (3.0)
Post-Rotate-135	-16.9 (2.4)	-15.3 (2.4)	-15.8 (2.3)	-14.1 (2.6)	-14.0 (2.3)	-13.1 (2.6)	-14.8 (2.2)	-14.0 (2.2)	-13.4 (2.2)	-13.7 (2.1)

Combined 45	-9.4 (1.8)	-10.3 (2.0)	-10.5 (2.0)	-11.2 (2.2)	-11.4 (2.2)	-11.7 (2.2)	-11.8 (2.2)	-12.2 (2.1)	-12.5 (2.2)	-12.6 (2.2)
Combined 135	-14.6 (2.3)	-14.6 (2.4)	-14.4 (2.2)	-13.3 (2.3)	-13.3 (2.3)	-12.8 (2.5)	-13.1 (2.3)	-12.5 (2.3)	-12.7 (2.3)	-12.5 (2.3)
